# Acute Promyelocytic Leukemia in Children: A Model of Precision Medicine and Chemotherapy-Free Therapy

**DOI:** 10.3390/ijms22020642

**Published:** 2021-01-11

**Authors:** Carmelo Gurnari, Maria Teresa Voso, Katia Girardi, Angela Mastronuzzi, Luisa Strocchio

**Affiliations:** 1 Department of Pediatric Hemato-Oncology and Cell and Gene Therapy, Istituto di Ricovero e Cura a Carattere Scientifico Bambino Gesù Children’s Hospital, 00165 Rome, Italy; CARMELOGURNARI31@GMAIL.COM (C.G.); katia.girardi@opbg.net (K.G.); angela.mastronuzzi@opbg.net (A.M.); 2 Department of Biomedicine and Prevention, University of Rome Tor Vergata, 00133 Rome, Italy; voso@med.uniroma2.it; 3 Immunology, Molecular Medicine and Applied Biotechnology, University of Rome Tor Vergata, 00133 Rome, Italy; 4 Department of Translational Hematology and Oncology Research, Taussig Cancer Institute, Cleveland Clinic, Cleveland, OH 44195, USA; 5 Laboratorio di Neuro-Oncoematologia, Fondazione Santa Lucia, 00179 Rome, Italy

**Keywords:** acute promyelocytic leukemia, children, arsenic trioxide, all-trans retinoic acid

## Abstract

Acute promyelocytic leukemia (APL) represents a paradigm of precision medicine. Indeed, in the last decades, the introduction of all-trans retinoic acid (ATRA) and arsenic trioxide (ATO) completely revolutionized the therapeutic approach to this previously highly fatal disorder. This entirely chemotherapy-free treatment, which provided excellent survival rates, has been initially validated in adults and, recently, translated in the pediatric setting. This review summarizes currently available data on the use of ATRA and ATO combination in pediatric APL, providing a particular focus on peculiar issues and challenges, such as the occurrence of *pseudotumor cerebri* and death during induction (early death), as well as the advantage offered by the ATO/ATRA combination in sparing long-term *sequelae*.

## 1. Epidemiology and Biology of Acute Promyelocytic Leukemia in Childhood 

Acute promyelocytic leukemia (APL) is a unique subtype of acute myeloid leukemia (AML) accounting for 5–10% of all pediatric AML cases [1]. 

A higher incidence has been described in Italy, Spain, China, and Latin America, with the highest proportions of APL (up to 50% of all AML cases) reported in Nicaragua and Argentina [2,3,4]. The incidence becomes higher with age with a peak in the fourth decade, while APL is particularly rare in infants (median age of pediatric cases is 9–12 years) [5,6]. Data from a population-based study of SEER (Surveillance, Epidemiology, and End Results Program) on demographics and epidemiology of 1397 patients with APL diagnosed between 1975 and 2008 in the United States showed an incidence of 0.06 per 100,000 persons/year in the pediatric population (aged ≤ 20 years) [7]. Moreover, APL occurs equally in both males and females, and obesity has been recognized as a possible risk factor [8].

Considered to be a rare disease, childhood APL has been traditionally treated borrowing adult protocols and, only in the last two decades, dedicated pediatric protocols have been designed.

After the first description in 1957 [9], in 1977 Rowley and colleagues identified the typical balanced chromosomal translocation t(15;17)(q22;q21). This represents a pathogenetic hallmark of APL and is detected in 95–98% of cases [10]. This translocation, joining the promyelocytic leukemia (PML) gene on chromosome 15 to the retinoic acid receptor alpha (RARA) gene on chromosome 17, leads to an oncogenic fusion gene (PML-RARA) [11]. In particular, the breakpoint of the RARA gene is located in its second intron, while three different breakpoints of the PML gene (intron 6, exon 6, and intron 3) identify the most common breakpoint cluster regions (bcr-1, -2, and -3, respectively), corresponding to different PML-RARA isoforms [12]. While bcr-1 (also known as “long” or “L” isoform) and bcr-3 (also known as “short” or “S” isoform) account for the majority of APL cases (90–95%), bcr-2 represents a more rare variant (also called “V”) [13] and no particular differences (apart from those related to ethnicity) are known as to the different frequency of these isoforms between children and adults [14,15].

**Figure 1:**
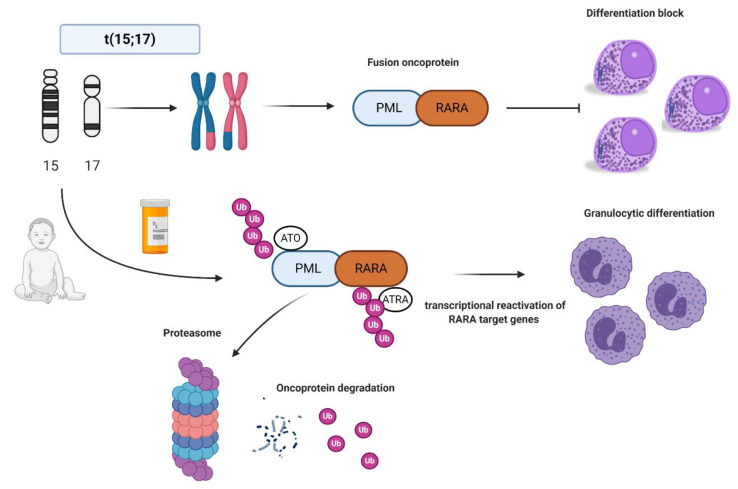
Acute promyelocytic leukemia (APL) biology and all-trans retinoic acid/arsenic trioxide (ATRA/ATO) synergistic treatment. APL is the result of the balanced translocation between promyelocytic leukemia (PML) gene on chromosome 15 and retinoic acid receptor alpha (RARA) on chromosome 17 leading to the fusion oncoprotein PML-RARA and myeloid differentiation block at promyelocyte stage. The combination of ATRA and ATO leads to ubiquitination and, ultimately, to proteasome degradation of the fusion protein, reactivation of the transcription of RARA target genes, and finally granulocytic differentiation. Images were generated using BioRender.

In normal conditions, PML interacts with p53 and it is critical for cellular senescence and tumor suppression activities, while RARA interacts as a heterodimer with RXR and regulates transcription factors important for myeloid differentiation [16]. In APL, the PML-RARA fusion oncoprotein is responsible for the myeloid differentiation block at the promyelocyte stage [17]. The combination of all-trans retinoid acid (ATRA) and arsenic trioxide (ATO) allows the proteasomal degradation of the oncoprotein via its ubiquitination and finally reactivates the transcription of RARA target genes [18,19,20] (Figure 1). Other mechanisms of action include the recruitment of caspases, the production of reactive oxygen species, and the reformation of PML-nuclear bodies, whose architecture is disrupted by PML-RARA fusion protein with critical consequences for normal cellular functions, such as self-renewal [21].

## 2. Diagnostics, Clinical Features, and Risk Assessment

As aforementioned, the classical hallmark of APL is the cytogenetic translocation t(15;17), present in 95–98% of cases, and detectable by the specific fluorescence in situ hybridization (FISH) probes, and more rapid molecular biology techniques such as reverse transcriptase polymerase chain reaction (RT-PCR). However, a small percentage of cases presents different RARA fusion partners, such as nucleophosmin (NPM1, 5q35), signal transducer, and activator of transcription (STAT5B, 17q21), factor interacting with PAPOLA and CPSF1 (FIP1L1, 4q12), promyelocytic leukemia zinc finger (PLZF, now known as ZBTB16, 11q23) and nuclear matrix-mitotic apparatus protein 1 (NUMA1, 11q13) [22,23,24,25,26]. These rare cases of “APL variant” are characterized by the same clinical picture of classic APL but different sensitivity to ATRA/ATO combination [27,28] (Table 1).

The presence of Auer rods and hypergranular promyelocyte blast cells is typical of APL. Frequently, the bone marrow classical morphology reveals promyelocytes with abundant Auer rods in multiple clusters (the so-called “faggot cells”). In children, the higher incidence of the microgranular variant (M3v) is controversial. Indeed, in pediatric APL, a higher incidence of the M3v has been reported (30% of pediatric APL vs. 15% in the adult population [29]), but these data have not been confirmed by other studies [29,30,31]. Although the confirmation of the diagnosis is typically based on PCR-based techniques, the immunophenotype study of classic APL is characteristic with the absence or low expression of HLA-DR, CD34, CD11a, and CD11b, bright expression of CD33, and heterogeneous expression of CD13 and CD117 in most cases. MPO is always positive with a high side scatter [29]. Conversely, M3v is frequently associated with the expression of CD2 and CD34 [29,32].

Despite its inclusion in the last European LeukemiaNet (ELN) 2017 recommendations under the umbrella of favorable/low-risk AML subtype together with the core-binding factor (CBF) leukemias, APL represents a unique disease with peculiarities in terms of clinical features and risk assessment [33].

The classical onset with a life-threatening coagulopathy characterized by severe and extended hemorrhages makes APL one of the most challenging hematological emergencies, which can lead the patient to death unless an adequate treatment is started [34]. APL blasts express fibrinolytic proteins, procoagulant factors, and proteolytic enzymes and have an increased ability to adhere to the vascular endothelium and secrete inflammatory cytokines, which ultimately stimulate the expression of prothrombotic proteins by leukocytes and endothelial cells [35]. This thrombo-hemorrhagic disturbance creates a clinical picture resembling disseminated intravascular coagulation, which can have different degrees of severity, ranging from laboratory alterations to life-threatening situations. The problem of early deaths (ED), defined as deaths occurring in the first 30 days after diagnosis, is, nowadays, one of the hindrances towards the cure of APL patients [36]. ED have been described to occur in approximately 10% of APL patients treated with ATRA plus chemotherapy in large cooperative trials; however, registry studies suggest a possible discrepancy with the “real world” situation, in which an ED rate up to 29% has been reported [37]. The leading causes of death are intracranial (as high as 70% of cases) followed by intrapulmonary hemorrhage [38,39]. In children, serious bleeding episodes have been reported in up to 15% of cases, and up to 10% of fatal outcomes in some series [40,41,42]. ED has been associated with a high white blood cells (WBC) count. In fact, WBC count at diagnosis is the strongest predictor of outcome and relapse in APL, so that patients are stratified according to the Sanz-risk criteria as standard risk (SR) and high risk (HR), based on the presence of less or more than 10,000/μL WBC, respectively [43].

## 3. Clinical Studies of APL in Children: Towards a “Chemotherapy-Free” Approach

### 3.1. The All-Trans Retinoic Acid (ATRA)-Era

First introduced over three decades ago, ATRA was first used as a single agent, and thereafter in combination with anthraycline-based chemotherapy to improve patient outcomes and minimize the risk of relapse [44,45,46]. The Italian GIMEMA-AIEOP AIDA-0493 trial was a seminal study evaluating the combination of ATRA with chemotherapy in newly diagnosed children with APL [30]. The treatment consisted of ATRA and idarubicine (AIDA) as induction therapy, followed by three athracyclines-based consolidation courses plus a maintenance phase in which patients were randomized into the following four arms: 6-mercaptopurine and methotrexate, ATRA alone, alternating chemotherapy and ATRA, and no further therapy.

During induction, ATRA was administered at a dose of 25 mg/m^2^/die in two divided doses until the achievement of complete remission, or 90 days, instead of 45 mg/m^2^/die employed in the adult population, due to a higher incidence of *pseudotumor cerebri* (PTC) reported to occur in the pediatric setting (see the dedicated section below). This dosage has been proven to be effective in previous adult dose-reduction studies and, subsequently, it has been used in all pediatric studies (unless otherwise specified in this manuscript) [47]. The majority (96%) of patients achieved complete remission (CR) with clearance of the PML-RARA transcript by RT-PCR at the end of consolidation phase. Overall survival (OS) and event-free survival (EFS) were 89% and 76% at more than 10 years, and the strongest outcome predictor was WBC counts > 10.000/μL (EFS of 59% vs. 83% in HR and SR patients, respectively). ED were registered in four cases, all included in the Sanz-HR category and, of note, three out of four patients died because of hemorragic complications. PTC occurred in 10 cases, a definite differentiation syndrome was registered in two patients, while, in the other six children, this complication was diagnosed as “indeterminate”. The differentiation syndrome (DS) is a complication that follows the initiation of ATRA treatment and occurs in about 5% to 20% of children, especially if treated with the ATRA/ATO combination [48,49]. The clinical picture of this life-threatening condition, contributing to ED during induction phase, is characterized by fever, dyspnoea, weight gain, pleuro-pericardial effusions, hypotension, and renal failure probably due to differentiating promyelocytes causing endothelial damage [50]. Risk factors include hyperleukocytosis and obesity, while treatment strategies include ATRA or ATO withdrawal, diuretics, and dexamethasone [8,51]. Prophylactic steroids from the first day of induction and hydroxyurea for the management of leukocytosis have been introduced for the prevention of this condition now diagnosed according to Montesinos’ criteria, which help the recognition of DS, allowing prompt initiation of treatment [52].

Several trials have confirmed the results of the Italian AIDA-0493 study, demonstrating that the use of ATRA plus chemotherapy could improve disease-free survival (DFS) as compared with chemotherapy-only regimens [53]. Moreover, a multicenter study analyzing data of two consecutive European trials (APL 93 and APL 2000) showed that children aged less than five years were at a higher risk of relapse with cumulative incidence of relapse (CIR) of 52% as compared with 18% of children aged 5–18 years (*p* = 0.006) [31]. Following the AIDA-0493 study, Italian pediatric patients were enrolled in a second multicenter GIMEMA-AIEOP trial, the AIDA-2000, which consisted of the same induction followed by a risk-adapted consolidation (three less intensive anthracycline-based courses plus ATRA for SR patients, three polychemotherapy consolidation courses plus ATRA for HR patients). However, the cumulative anthracycline dose was maintained as it was for the previous AIDA-0493 trial, with no differences for SR and HR groups. In an attempt to reduce cardiotoxicity and therapy-related second neoplasms, especially important for children who have a long life expectancy, the following international ICC-APL-01 study (International Consortium for Childhood APL) was designed to explore the outcomes of newly diagnosed children with APL, treated with reduced cumulative anthracycline doses and a risk-stratified consolidation treatment based on the Sanz-risk criteria [1].

This study enrolled 258 patients treated in several countries of Europe and South America receiving ATRA and idarubicine-based induction followed by two or three consolidation blocks according to the risk category, plus a two-year long maintenance therapy. This protocol allowed a remarkable anthracycline dose reduction from the 650 mg/m^2^ of AIDA-0493 and -2000 trials, to 355 mg/m^2^ and 405 mg/m^2^ of the ICC-APL-01 study for SR and HR APL, respectively. This reduction in the anthracycline cumulative dose did not affect outcomes, which were similar to the previous studies, with CR achieved in 97% of cases, and five-year OS and EFS of 98.4% and 89.4% in SR patients, and 84.3% and 74.2% in HR patients, respectively (*p* = 0.002 and *p* = 0.043, respectively). The most common adverse event was febrile neutropenia due to myelosuppression (42% in induction and 60% in consolidation phases), still remaining a common side effect of chemotherapy-based regimens, and a hurdle to face in this setting of patients [1].

#### A Peculiarity of Childhood APL: *Pseudotumor Cerebri*

**Figure 2:**
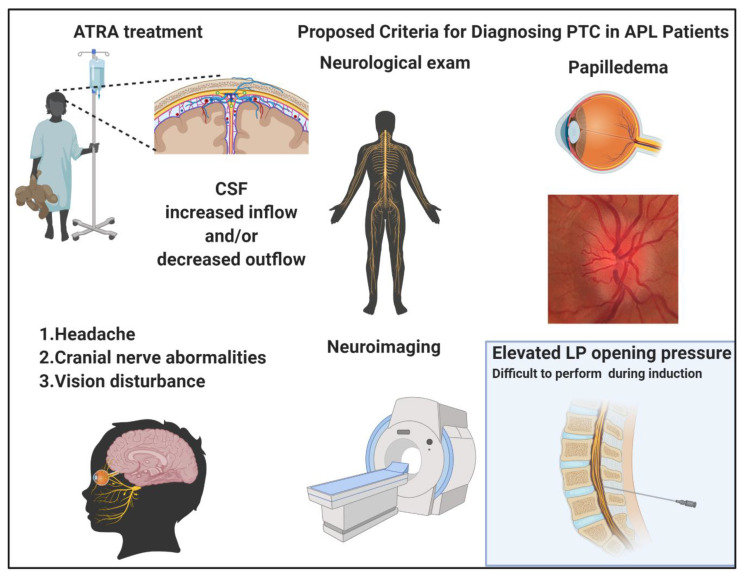
*Pseudotumor cerebri* (PTC) in acute promyelocytic leukemia (APL). All-trans retinoic acid (ATRA) treatment creates cerebrospinal fluid (CSF) imbalances, increasing the inflow and/or decreasing the outflow. The clinical picture is characterized by headache, cranial nerve, and vision abnormalities. The diagnostic criteria proposed for PTC include a normal neurological exam with exclusion of cranial nerve lesions, presence of papilledema, neuroimaging indicating increased intracranial pressure and, whenever possible to be performed, elevated lumbar puncture opening pressure with normal CSF composition. Images were generated using BioRender.

PTC is a typical complication of ATRA administration registered in about 3% of patients overall, and in up to 15% of pediatric patients [54]. Indeed, in the pediatric setting, the ATRA dose has been lowered to 25 mg/m^2^/die in order to avoid this complication. The clinical picture is characterized by headache, cranial nerves dysfunction, and vision disturbances until permanent blindness, in 10% of cases developing this complication. PTC may be a primary disorder common in obese, female patients of childbearing age, or also secondary to cerebral venous abnormalities or drug administration as in the case of ATRA. (Figure 2) The mechanism of ATRA neurotoxicity is similar to vitamin A intoxication, with an increased production of cerebrospinal fluid (CSF) and alteration of the lipid structure of the arachnoid villi with impairment of CSF reabsorption [55,56]. The revised diagnostic “Dandy criteria” for PTC require the presence of papilledema, a normal neurological exam except for cranial nerve abnormalities, elevated lumbar puncture (LP) opening pressure (≥250 mmHg or ≥280 mmHg for non-obese children), and normal neuroimaging [57]. However, it is often challenging to perform an LP in a patient with APL especially in the induction phase, because of the profound coagulopathy and the thrombocytopenia that these patients inevitably experience. Moreover, it has been reported that, in almost half of APL patients, the opening pressure does not meet the diagnostic criteria for PTC. A recent case report showed that a particular MRI-based neuroimaging may help the diagnosis of these cases without performing a LP [58]. In particular, the presence of an empty sella, as well as the distension of the perioptic subaracnoid space and of the optic nerve sheath were all indirect signs of elevated intracranial pressure. To overcome the difficulty of performing an LP in this setting of patients, new specific diagnostic criteria for ATRA-related PTC were proposed by Coombs et al., after a metanalytic study of 35 cases reported in the literature at the time of the analysis. In this study, the median age of PTC diagnosis was 18 years with a median time of PTC onset of 14 days from diagnosis (when occurring during induction) [59,60]. ATRA discontinuation was sufficient for the resolution of PTC in around 30% of cases, while, in other cases, the signs and symptoms improved after therapeutic LP or other therapies, including mannitol, acetazolamide, and topiramate. Of note, topiramate has been used in patients failing acetazolamide, with good results in a report where the authors registered a surprisingly high incidence of PTC (50%, five out of 10 patients) [61]. When looking at patients’ characteristics and the ATRA/ATO treatment schedule, ATRA was used at 45 mg/m^2^/die and four out of five cases had a body mass index (BMI) higher than 28 kg/m^2^, all known risk factors for APL complications. Acetazolamide is a carbonic anhydrase inhibitor used for reducing CSF production, while the topiramate mechanism of action seems to be related to its activity similar to anhydrase type II and IV receptors inhibition and migrainolytic effects, via augmentation of baseline GABAergic activity.

### 3.2. The ATO-Era

#### 3.2.1. Translating the Lesson of the APL0406 Protocol

In the seminal phase three Italian-German study APL0406, the combination of ATRA and ATO led to impressive cure rates and survival outcomes in SR adult patients with APL, paving the way for totally chemotherapy-free protocols [62]. The treatment, which was compared in a randomized way to the classical AIDA regimen, demonstrated a higher EFS in patients given the ATO-ATRA combination, as a result of the more efficacious antileukemic activity. This long-lasting efficacy, recently confirmed in a publication reporting updated results, provided the basis for the elimination of the maintenance phase [63,64]. Moreover, the results of this study documented that the ATRA/ATO combination was of relevant benefit also in FLT3-ITD mutated cases with the abrogation of the role of this negative prognostic factor linked to higher WBC counts, and present in up to 40% of both adult and pediatric APL cases [65]. As a result, in the same years, multiple studies were conducted in order to translate the adult experiences in children and try to lower anthracyclines exposure while achieving the best antileukemic efficacy.

First used for relapsing patients, ATO showed great efficacy even as a single agent for induction and consolidation therapy in newly diagnosed children with APL. George et al. demonstrated that ATO alone can be as effective as ATRA plus chemotherapy in newly diagnosed children with APL, with CR of 91% and estimated five-year OS and EFS of 91% and 81%, respectively [66,67,68]. Another Chinese study included 19 children aged less than 15 years treated upfront with ATO alone during induction and post-remission therapy [69]. CR was achieved in 89.5% of cases with two episodes of ED due to intracranial hemorrhage. Overall survival and EFS were 83.9 and 72.7% at five years, respectively. Of note, the only patient who did not respond to ATO had additional karyotype abnormalities, while two patients with ED were classified as HR. Post-remission ATO administration was completed over three years (different from George et al., where ATO was administered for eight months), and it is noteworthy that this study provided important insights into arsenic elimination kinetics [68,69]. In fact, urine and nails/hair arsenic concentrations were studied in nine of the 19 treated patients and were compared with the patients’ parents as controls. The results of this analysis showed that arsenic concentration in all the specimens of patients who completed the treatment for at least 24 months were not higher than those found in the controls (*p*_urine_ = 0.556, *p*_hair_ = 0.542, *p*_nails_ = 0.436) and, when looking at the urine compartment, the arsenic levels were below the safe limit of the U.S. Agency for Toxic Substances and Disease Registry (ARTSDR) [69].

Following these encouraging results, the North American APL intergroup study CALGB 9710 randomized patients aged 15–18 years to two consolidation courses with ATO after a classic ATRA/chemotherapy-based induction, showing a lower relapse risk at five years for those receiving ATO (0% vs. 44% for patients who did not receive the drug, *p* = 0.02) [70]. Another study from the same group investigated the possibility of a lower anthracycline dose in 101 pediatric patients with APL (66 SR and 35 HR) introducing ATO during the consolidation phase. This led to a lower cumulative anthracycline dose of 335 and 385 mg/m^2^ for SR and HR patients (similar to the aforementioned dose of the ICC-APL-01 study which did not include ATO) maintaining excellent survival outcomes, with a three-year EFS of 91% [48]. The same conclusions were drawn by a Chinese randomized trial, which clearly demonstrated that cytarabine could also be omitted when ATO was used for two courses in an attempt to avoid greater myelosuppression when ATO and ATRA were used in combination with chemotherapy, and with no differences in terms of survival outcomes [71].

#### 3.2.2. The Use of Upfront ATRA/ATO Combination in Children

Up to now, a frontline treatment approach based on the ATO-ATRA combination has been explored in a limited number of pediatric patients. A series of 43 children were first treated with ATRA/ATO combination in both induction and consolidation phases in a Chinese randomized trial [72]. However, the consolidation courses still contained chemotherapy, and a maintenance phase with ATRA and mercaptopurine was administered. The first European case series of 11 children with SR-APL was, then, reported by the AML-Berlin-Frankfurt-Münster (BFM) group. In this study, the ATRA/ATO treatment schedule was modified from the APL0406 protocol with a delayed start of ATO (given on day 10) in order to avoid the risk of hyperleucocytosis, and the inclusion of a one-week break of ATRA after the first 14 days of treatment in order to avoid a rapid decrease in plasma drug concentration (continuous schedule) and provide repetitive periods of higher plasma drug exposure (intermittent schedule) [73]. Moreover, in three cases, patients received additional chemotherapy for uncontrolled leukocytosis [74]. After the BFM experience, our group published the first case series (18 children) of children with newly diagnosed APL treated with a chemotherapy-free approach consisting of ATO plus ATRA for both induction and consolidation therapy, based on the protocol scheme described for SR adult patients in the APL0406 trial, with the only difference of a lower ATRA dose for the aforementioned risk of PTC [75]. Of note, two out of 18 patients were classified as HR and none of the children received any chemotherapeutic agents other than hydroxyurea for the control of leukocytosis, registered in 62% of cases during induction. All patients achieved CR and were PML/RARA negative at the end of the third consolidation course. Toxicities were transient and manageable and included one patient with a suspected DS, and two patients presenting PTC. Another recent French study confirmed our results in a case series of 21 children aged less than 16 years [76]. Of note, in the last two case series, which used a totally chemotherapy-free regimen resembling the APL0406 trial, the typical QTc prolongation observed with ATO was registered in 9.5% and 16.6% of cases, in the French and Italian experiences, respectively. However, a difference was registered in the incidence rate of hepatic toxicity, with a 50% in the Italian report and no cases in the French experience [75,76].

The new International ICC-APL-02 and North American COG-AAML1331 trials, aimed at evaluating the use of ATRA/ATO combination upfront in newly diagnosed patients with SR-APL, with the addition of few low-dose athracyclines for the HR group, are actively recruiting. In particular, similar to the ongoing adult APOLLO trial (EudraCT 2015-001151-68), the ICC-APL-02 trial is designed as a complete chemotherapy-free treatment also for patients allocated to the HR group; these children are candidate to receive ATRA/ATO according to the classic schedule, plus gentuzumab-ozogamicin (GO) on the second and fourth days of induction at 3 mg/m^2^ with the rationale of controlling/preventing hyperleukocytosis together with hydroxyurea. A possible idarubicine single dose (12 mg/m^2^) is allowed in case the patient is unable to receive the first dose of GO within the first 72 h from diagnosis for logistic reasons. Moreover, only HR patients are candidate to receive intrathecal therapy at the beginning of the first and third consolidation block with methotrexate, methylprednisolone, and cytarabine at doses chosen according to the patients’ age. (Eudract no. 2017-002383-40).

#### 3.2.3. Oral ATO Formulations in Children

Finally, a recent Chinese trial showed promising results in pediatric APL, with an oral formulation of ATO named Realgar-Indigo naturalis formula (RIF), an arsenic-containing compound deriving from natural Chinese medicine [77]. Following the successful trials with noninferiority results as compared with the intravenous formulation, the Chinese Food and Drug Administration approved RIF in 2009 for the treatment of adult APL [78]. The better pharmacokynetics profile of the oral formulation led to lower cardiac and hepatic toxicities, and to a “largely home-based treatment protocol”, at least during the post-induction phases. This latter aspect is of great importance if we consider the pediatric population, the obvious psychological implications of fewer hospital stays for this setting of patients, and the economic costs for health systems and families. In particular, the SCCLG-APL trial enrolled 82 patients to either receive ATO (*n* = 42) or RIF (*n* = 40) [77]. The estimated OS and EFS were 100% for both arms, with significantly less hospital stay in the RIF group and milder toxicities. However, it has to be noted that in the induction regimen of HR patients, the Chinese group used a short course of mitoxantrone administered, in both arms, on day three or on days two or four at 7 mg/m^2^/day. Moreover, intravenous ATO was given over 12 h instead of the typical 2 h as in the APL0406 trial, because of the evidence that longer ATO exposure may reduce differentiation and promote apoptosis of APL cells [79]. Finally, lower cardiac toxicity has been reported during RIF administration [80,81].

## 4. Pitfalls and Hurdles of Childhood APL: Relapses and Long-Term *Sequelae*

With the use of ATRA and ATO combination, APL relapses have become a rare entity, also in children [82]. Molecular studies on relapsed APL have been typically conducted using adult patient samples and helped to identify the molecular landscape of ATRA-resistant clones retaining self-renewal activities and the contribution of other genes, such as WT1, FLT3, and PML mutations [83,84]. No specific trials have systematically addressed the appropriate therapy and management of relapses and future prospective trials are probably not feasible, especially in children, because of the rarity of the event. However, sporadic case reports showed that ATO may be used for achieving a second CR in pediatric cases, underlying that ATO resistance is almost absent, with the exception of the aforementioned cases carrying PML mutations, with A216V as the predominant lesion [85]. Moreover, GO, an anti-CD33 humanized monoclonal antibody linked to the cytotoxic agent calicheamicin, has also been used with successful results and is approved for children under two years old, at doses lower than 6 mg/m^2^ [86]. Therefore, patients achieving molecular remission may proceed to autologous stem cell transplant, while patients unable to clear the molecular transcript should receive an allogeneic stem cell transplant [82]. Finally, tamibarotene, a synthetic retinoid analog, can overcome ATRA resistance thanks to higher PML-RARA binding affinity, and appears to be promising in the relapsed/refractory setting [87].

Another important problem in the field of childhood APL is long-term *sequelae*. If the occurrence of cardiac toxicity seems to be avoided with the newer anthracycline-free regimens, prolonged arsenic exposure may lead to skin lesion (hyperpigmentation, keratosis, and squamous cell carcinoma), hypertension, diabetes mellitus, neurologic effects, and urinary tract cancers (mainly involving the bladder) [88]. The results of arsenic elimination pharmacokinetics of the study by Zhou et al. and Li et al. seem to be promising, at least demonstrating that arsenic does not last more than 24 months above the safety threshold set by the regulatory agencies [69,71]. Moreover, the post-remission treatment is now based on shorter ATO exposure (less than eight months as compared with the three year post-remission strategy of the first Chinese study). Finally, in children, arsenic might cause an alteration of growth and development, although available data are immature for evaluating long-term toxicities and we need to wait for the results of the ongoing studies. However, the recent data of the oral formulation, showing a better toxicity profile and simpler administration modality as compared with the intravenous formulation, seem to be particularly attractive for the younger population [77].

## 5. Conclusions and Future Perspectives

APL is a paradigmatic example of modern precision medicine. The introduction of the ATRA/ATO combination has led APL from being an extremely lethal disease to a highly curable condition, even with completely chemotherapy-free approaches. The ongoing pediatric ATRA/ATO combinatory trials represent a breakthrough in the treatment of childhood APL, with an emblematic shift towards lower exposure to chemotherapeutic agents, characterized by both acute and long-term side effects. The new oral arsenic formulations may allow a better compliance in younger patients and will further minimize the potential side effects of intravenous agents. Finally, and importantly, the safer toxicity profile of oral vs. intravenous arsenic formulations may also represent a key for reducing hospital stays with important implications in psychological aspects and quality of life of children facing their oncologic journey.

## Figures and Tables

**Table 1:** ATRA/ATO sensitivity data of the various RARA rearrangements mentioned in the text.

RARA Rearrangements	Cytogenetic Translocations	ATRA Sensitivity	ATO Sensitivity
PML-RARA	t(15;17)(q22;q21)	S	S
FIP1L1-RARA	t(4;17)(q12;q21)	I	NA
NPM1-RARA	t(5;17)(q35;q21)	S	NA
NuMA-RARA	t(11;17)(q13;q21)	S	NA
STAT5b-RARA	t(17;17)(q21;q21)	R	R
ZBTB16-RARA	t(11;17)(q23;q21)	R	R

R, resistant; I, intermediate sensitivity; S, sensitive; NA, data not available.

## Data Availability

Not applicable.
